# Cell division cycle proteinising prognostic biomarker of breast cancer

**DOI:** 10.1042/BSR20191227

**Published:** 2020-05-05

**Authors:** Lin Cheng, Yu-Zhou Huang, Wei-Xian Chen, Liang Shi, Zhi Li, Xu Zhang, Xin-Yuan Dai, Ji-Fu Wei, Qiang Ding

**Affiliations:** 1 Jiangsu Breast Disease Center, The First Affiliated Hospital with Nanjing Medical University, 300 Guangzhou Road, Nanjing 210029, China; 2 Department of Breast Surgery, The Affiliated Changzhou No. 2 People’s Hospital of Nanjing Medical University, 29 Xinglong Lane, Changzhou 213003, China; 3 Research Division of Clinical Pharmacology, The First Affiliated Hospital with Nanjing Medical University, 300 Guangzhou Road, Nanjing 210029, China

**Keywords:** Bioinformatics analysis, Breast cancer, CDC20, Prognosis

## Abstract

Cell division cycle protein (CDC20) has been observed to be expressed higher in various kinds of human cancers and was associated with poor prognosis. However, studies on role of CDC20 in breast cancer are seldom reported till now, most of which are not systematic and conclusive. The present study was performed to analyze the expression pattern, potential function, and distinct prognostic effect of CDC20 in breast cancer using several online databases including Oncomine, bc-GenExMiner, PrognoScan, and UCSC Xena. To verify the results from databases, we compared the mRNA CDC20 expression in breast cancer tissues and adjacent normal tissues of patients by real-time PCR. We found that CDC20 was expressed higher in different types of breast cancer, comparing with normal tissues. Moreover, the patients with a more advanced stage of breast cancer tended to express higher level CDC20. CDC20 was expressed higher in breast cancer tissues than normal tissues from patients in our hospital, consistent with the results from databases. Estrogen receptor (ER) and progesterone receptor (PR) status were negatively correlated with CDC20 level. Conversely, Scarff–Bloom–Richardson (SBR) grade, Nottingham prognostic index (NPI), epidermal growth factor receptor-2 (HER-2) status, basal-like status, and triple-negative status were positively related to CDC20 expression in breast cancer patients with respect to normal individuals. Higher CDC20 expression correlated with worse survival. Finally, a positive correlation between CDC20 and Targeting protein for Xenopus kinesin-like protein 2 (TPX2) expression was revealed. CDC20 could be considered as a potential predictive indicator for prognosis of breast cancer with co-expressed TPX2 gene.

## Introduction

Breast cancer is the most common malignant tumor and remains a major cause of deaths in women [1]. With the development of treatment, including surgery, chemotherapy, radiotherapy, endocrine therapy, and target therapy, both disease-free survival and overall survival (OS) of breast cancer have been significantly improved. However, after systemic therapies, there are still some patients that died of breast cancer, especially for advanced breast cancer. Breast cancer is related with inactivation of a large number of tumor suppressor genes and oncogenes [2]. As we know, biomarkers are reported as surrogates of the clinical features for predicting outcomes. It is important to identify more effective, sensitive, and specific biomarkers for the prognosis of patients with breast cancer [3].

The cell-division cycle consists of a series of complex processes which are regulated by numbers of cell cycle regulatory proteins [4]. The cell division cycle protein (CDC) 20 (CDC20), acting as a regulatory protein, is a target molecule in the cell-cycle checkpoint [5]. It is also a key E3 ligase, which can activate adenomatous polyposis coli (APC) [5]. In addition to regulating cell cycle, recent evidence has demonstrated that CDC20 also plays an important role in carcinogenesis and cancer progression, having the potential to become a promising therapeutic target [6]. CDC20 has been observed as expressed higher in different kinds of human cancers and was associated with poor prognosis such as, oral squamous cell carcinoma [7], gastric cancer [8], urothelial bladder cancer [9], colorectal cancer [10], lung cancer [11], and pancreatic cancer [12].

Recently, CDC20 has been demonstrated to act as an oncogene in breast cancer progression [13], However, studies on role of CDC20 in breast cancer are seldom reported till now, most of which are not systematic and conclusive. Moreover, the CDC20 expression’s prognostic significance is also uncertain. Therefore, it is imperative to recognize that further study is necessary to determine the oncogenic role of CDC20 in breast tumorigenesis.

In the present study, we performed a deep bioinformatics analysis of the clinical parameters and survival data related to CDC20 in breast cancer patients using several online databases in order to evaluate the prognostic significance of CDC20 gene in breast cancer treatment. Moreover, we used 22 pairs of breast tissues from breast cancer patients in our hospital to compare the expression of CDC20 gene between the cancer tissues and normal tissues by real-time PCR.

## Materials and methods

### ONCOMINE data-mining analysis

ONCOMINE (www.oncomine.org), an online web-based cancer database for RNA and DNA sequences, was used to facilitate data-mining of the transcriptional expressions of genes in 20 types of cancer [14]. Data used in the present study from ONCOMINE were updated in April 2019. Transcriptional expressions of CDC20 in cancer samples were compared with those in normal individuals using Student’s *t* test. Statistically significant values and fold change were demarcated as *P*-value ≤ 1E-4 and 2, respectively. Genes co-expressed with CDC20 were analyzed by using online Oncomine analysis tools.

### UALCAN

UALCAN (http://ualcan.path.uab.edu/) is a user-friendly, interactive web resource for analyzing transcriptome data of cancers from The Cancer Genome Atlas (TCGA) [15]. Data from UALCAN was updated in March 2019. The mRNA expression of CDC20 between breast cancer and normal tissues as well as different stages cancer was also detected using the UALCAN web portal (TCGA level 3 data).

### bc-GenExMiner v4.2

Breast Cancer Gene-Expression Miner v4.2 (bc-GenExMiner v4.2) is a statistical mining tool that incorporates 264 independent datasets and three classical mining functions: expression, prognosis, and correlation [16,17]. Expression data were analyzed with clinical parameters such as age, nodal status, estrogen receptor (ER), progesterone receptor (PR), epidermal growth factor receptor-2 (HER-2), Scarff–Bloom–Richardson (SBR) grade, and Nottingham prognostic index (NPI). Correlation of CDC20 and TPX2 was assessed using the correlation module.

### PrognoScan

The PrognoScan (http://www.prognoscan.org/) is an online database for assessing the biological relationship between gene expression and survival data including OS, distant metastasis-free survival, relapse-free survival (RFS), and disease-specific survival in breast cancer patients. The results are based on a collection of publicly available cancer microarray datasets [18]. Data used in the present study from PrognoScan were updated in April 2019. *P*-value, hazard ratio (HR), and 95% confidence intervals (CI) could be automatically calculated according to a certain gene expression. For statistical analysis and visualization, R packages (http://www.r-project.org) were used.

### UCSC Xena

The UCSC Xena (http://xena.ucsc.edu/) is a popular genomics browser that provides visualization and integration for analyzing and viewing the public data hubs [19]. The heat map and correlation between CDC20 and TPX2 were generated by data mining in TCGA Breast Cancer using the UCSC Xena browser.

### Breast tissue samples

Twenty-two pairs of breast tissue samples used in quantitative real-time PCR (RT-PCR) were obtained from the First Affiliated Hospital of Nanjing Medical University, China, between 2014 and 2016. The collection and use of the samples was reviewed and approved by the Institutional Ethics Committee of the First Affiliated Hospital of Nanjing Medical University.

### RNA isolation and RT-PCR analysis

Total RNA was isolated using TRIzol reagent (TaKaRa, Japan) and 1000 ng RNA was reverse-transcribed into cDNA using Primescript RT Reagent (TaKaRa, Japan). The RT-PCR was performed using FastStart Universal SYBR Green Master (Roche, Switzerland) in an RT-PCR instrument (Applied Biosystems, U.S.A.), and β-actin was used as an endogenous control. The following PCR primers were used:

CDC20 forward, 5′-GCACAGTTCGCGTTCGAGA-3′
CDC20 reverse, 5′-CTGGATTTGCCAGGAGTTCGG-3′
β-actin forward, 5′-GCTGTGCT ATCCCTGTACGC-3′
β-actin reverse, 5′-TGCCTCAGGGCAGCGGAACC-3′.

### Statistical analysis

RT-PCR were repeated in triplicate, unless otherwise specified. The data were analyzed using the SPSS 20.0 software (Chicago, U.S.A.). We analyzed the statistical significance of the differences between groups using Student’s *t* test, and a statistically significant difference was considered at the level of *P*<0.05.

## Results

### Increased expression of CDC20 gene in breast cancer patients’ tissues

**Figure 1:**
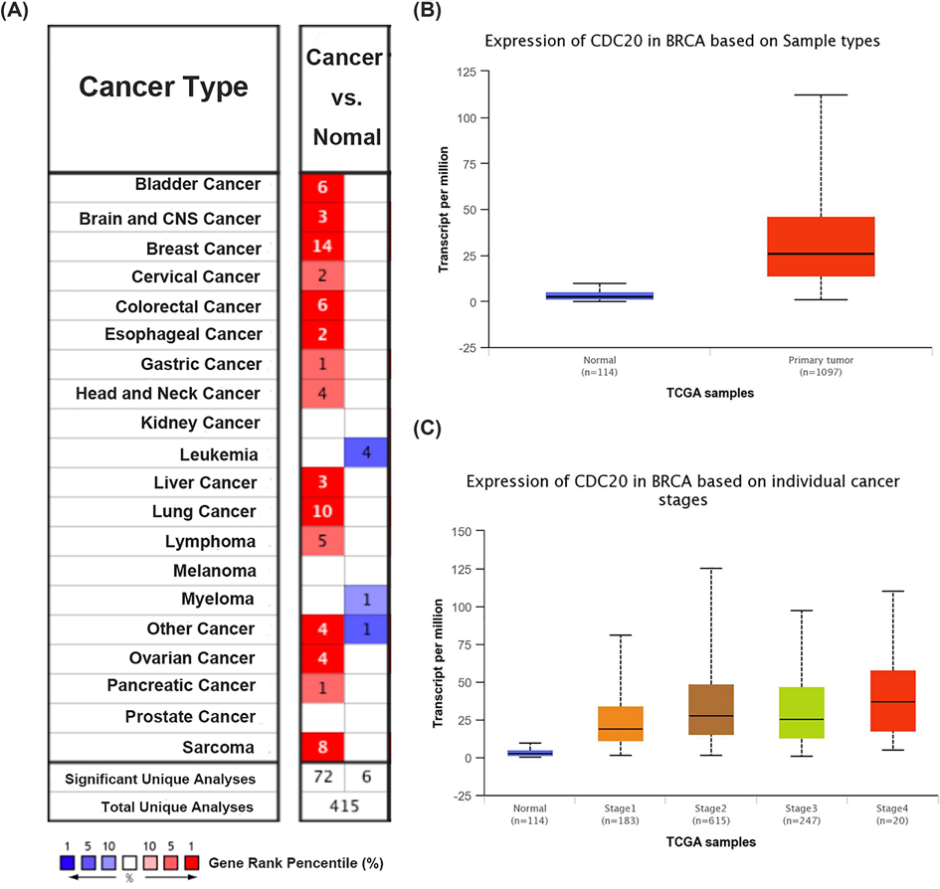
Increased expression of CDC20 gene in breast cancer tissues in public databases (**A**) Expression of CDC20 in 20 types of cancer vs. corresponding normal tissues using the Oncomine database with the threshold of fold change ≥ 2, *P*-value ≤1E-4, and gene rank ≥ top 10%. Red and blue, respectively, stand for the numbers of datasets with statistically significant (*P*<0.05) increased and decreased levels of CDC20 gene. (**B**) Higher mRNA CDC20 was expressed in breast cancer tissues than in normal tissues (*P*<0.05) using UALCAN website. (**C**) Patients with a more advanced stage of breast cancer tended to express higher levels of CDC20.

First, the expression of CDC20 gene in 20 types of cancer was measured and compared with normal tissues using the Oncomine online database (Figure 1A). We found that increased CDC20 (red) was observed in bladder cancer, brain and CNS cancer, cervical cancer, colorectal cancer, esophageal cancer gastric cancer, head and neck cancer, liver cancer, lung cancer, lymphoma, ovarian cancer, pancreatic cancer, sarcoma, and especially breast cancer, whereas, decreased level of CDC20 (blue) was observed in leukemia and myeloma. Consistently, using UALCAN website, we also found that higher mRNA CDC20 was expressed in breast cancer tissues than in normal tissues (Figure 1B, *P*<0.05). Next, we focused on whether mRNA expression of CDC20 was related to cancer stage in individual patients. As shown in Figure 1C, the results indicated that patients with a more advanced stage of breast cancer tended to express higher levels CDC20.

**Figure 2:**
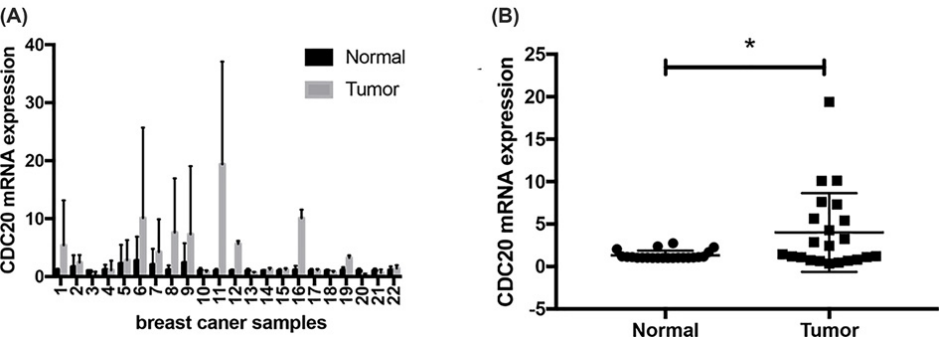
Increased expression of CDC20 gene in breast cancer tissues from patients in our hospital (**A**) CDC20 mRNA expression in 22 pairs of breast cancer and adjacent tissues. (**B**) Average expression level of RBMS2 mRNA in 22 pairs of breast cancer tissues and adjacent normal breast tissues. Breast cancer tissues had higher expression of CDC20 than adjacent breast tissues (*, P<0.05).

To verify the results above, we further compared the mRNA CDC20 expression in breast cancer tissues and adjacent normal tissues of patients in our hospital and found that CDC20 was expressed higher in breast cancer tissues, consistent with the results from databases (Figure 2, *P*<0.05).

**Figure 3:**
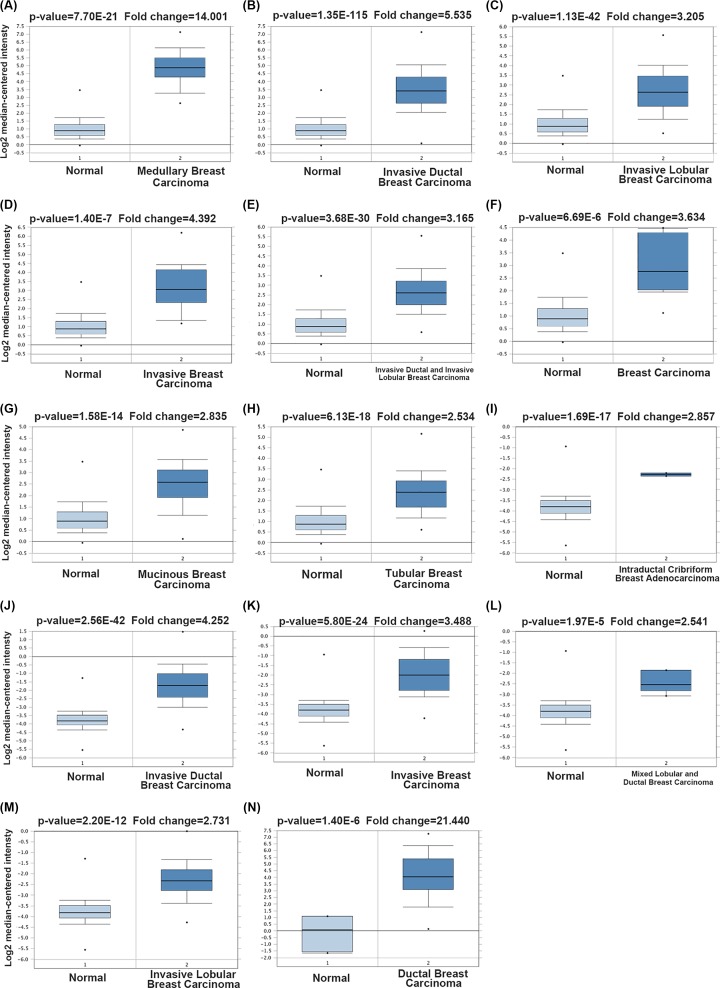
Box plot comparing CDC20 expression in normal individuals and breast cancer patients obtained from the Oncomine database Analysis is shown for medullary breast carcinoma (**A**), invasive ductal breast carcinoma (**B**), invasive lobular breast carcinoma (**C**), invasive breast carcinoma (**D**), invasive ductal and invasive lobular breast carcinoma (**E**), breast carcinoma (**F**), mucinous breast carcinoma (**G**), tubular breast carcinoma (**H**), intraductal cribriform breast adenocarcinoma (**I**), invasive ductal breast carcinoma (**J**), invasive breast carcinoma (**K**), mixed lobular and ductal breast carcinoma (**L**), invasive lobular breast carcinoma (**M**), ductal breast carcinoma (**N**).

Oncomine analysis also revealed that CDC20 was significantly expressed higher in medullary breast carcinoma, invasive ductal breast carcinoma, invasive lobular breast carcinoma, invasive breast carcinoma, invasive ductal and invasive lobular breast carcinoma, breast carcinoma, mucinous breast carcinoma, tubular breast carcinoma, intraductal cribriform breast adenocarcinoma, invasive ductal breast carcinoma, invasive breast carcinoma, mixed lobular and ductal breast carcinoma, invasive lobular breast carcinoma, ductal breast carcinoma with respect to normal individuals (Figure 3 and Table 1).

**Table 1:** The significant changes of CDC20 expression in transcription level between different types of breast cancer and normal tissues (ONCOMINE database)

Subtype of breast cancer	*P*-value	Fold change	Rank (%)	Sample	Reference
Medullary breast carcinoma	7.70E-21	14.001	1%	176	22522925
Invasive ductal breast carcinoma	1.35E-115	5.535	1%	1700	22522925
Invasive lobular breast carcinoma	1.13E-42	3.205	2%	292	22522925
Invasive breast carcinoma	1.40E-7	4.392	2%	165	22522925
Invasive ductal and invasive lobular breast carcinoma	3.68E-30	3.165	2%	234	22522925
Breast carcinoma	6.69E-6	3.634	3%	158	22522925
Mucinous breast carcinoma	1.58E-14	2.835	4%	190	22522925
Tubular breast carcinoma	6.13E-18	2.534	7%	211	22522925
Intraductal cribriform breast adenocarcinoma	1.69E-17	2.857	1%	64	TCGA
Invasive ductal breast carcinoma	2.56E-42	4.252	1%	450	TCGA
Invasive breast carcinoma	5.80E-24	3.488	2%	137	TCGA
Mixed lobular and ductal breast carcinoma	1.97E-5	2.541	3%	68	TCGA
Invasive lobular breast carcinoma	2.20E-12	2.731	3%	97	TCGA
Ductal breast carcinoma	1.40E-6	21.440	4%	47	16473279

### CDC20 expression and clinical parameters of breast cancer patients

**Figure 4:**
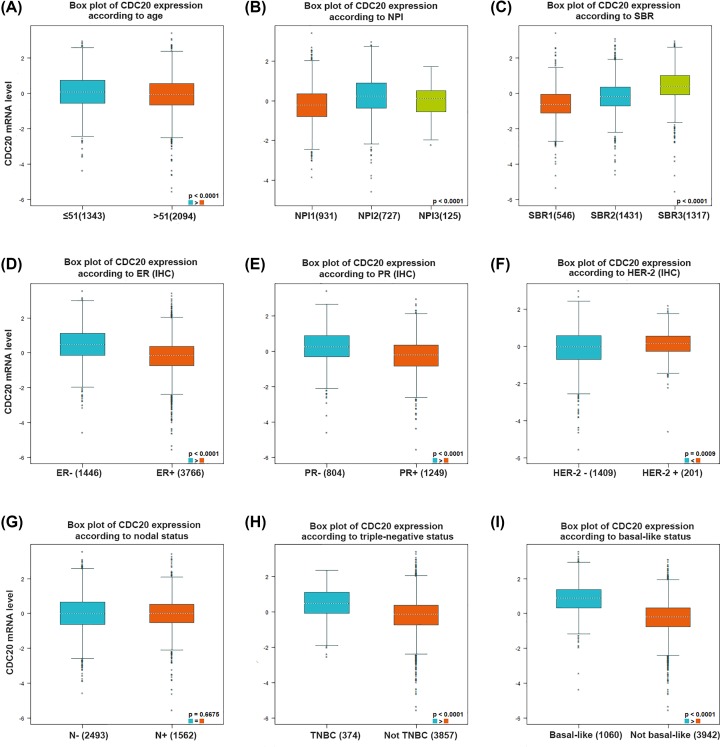
Box plot evaluating CDC20 expression among different groups of patients based on clinical parameters using the bc-GenExMiner 4.2 Analysis is shown for age (**A**), NPI index (**B**), SBR grade (**C**), ER (**D**), PR (**E**), HER-2 (**F**), nodal status (**G**), triple-negative status (**H**) and basal-like status (**I**).

Using bc-GenExMiner v4.2 software, we implemented Welch’s test to compare the abnormal expression of CDC20 among different groups of patients according to clinical pathological features. For age criteria, CDC20 was significantly elevated in ≤51-year group with respect to >51-year group (Figure 4A and Table 2). As we know, the SBR is a histological grade that evaluates tubule formation, nuclear characteristics of pleomorphism, and mitotic index [20]. Based on tumor size, lymph node stage, and tumor grade, the NPI is used to stratify patients into additional prognostic groups. The SBR grade and NPI index are two wonderful prognostic models for breast cancer [21]. More advanced SBR grade and NPI index were associated with higher CDC20 level (Figure 4B,C and Table 2). ER-positive or PR-positive breast cancer patients tended to express lower CDC20 gene compared with ER-negative or PR-negative patients (Figure 4D,E and Table 2). Patients with HER-2-negative status showed reduced expression of CDC20 than HER-2-positive patients (Figure 4F and Table 2). Regarding nodal status, there was no significant difference between positive and negative group (Figure 4G and Table 2). Moreover, CDC20 was significantly reduced in non-triple-negative and non-basal-like breast cancer patients compared with triple-negative and basal-like breast cancer patients (Figure 4H,I and Table 2).

**Table 2:** The relationship between mRNA expression of CDC20 and clinicopathological parameters of breast carcinoma

Variables	Number of patients	CDC20 mRNA	*P*-value
Age			
≤51	1392	↑	<0.0001
>51	2210		
Nodal status			
−	2493		0.6675
+	1562		
ER			
−	1446	↑	<0.0001
+	3766		
PR			
−	804	↑	<0.0001
+	1249		
HER2			
−	1409		0.0009
+	201	↑	
SBR			
1	546		<0.0001
2	1431	↑	
3	1317	↑	
NPI			
1	931		<0.0001
2	727	↑	
3	125	↑	
Basal-like status			
Not	3492		<0.0001
Basal-like	1060	↑	
Triple-negative status			
Not	3857		
TNBC	374	↑	<0.0001

### CDC20 expression and survival data of breast cancer patients

**Figure 5:**
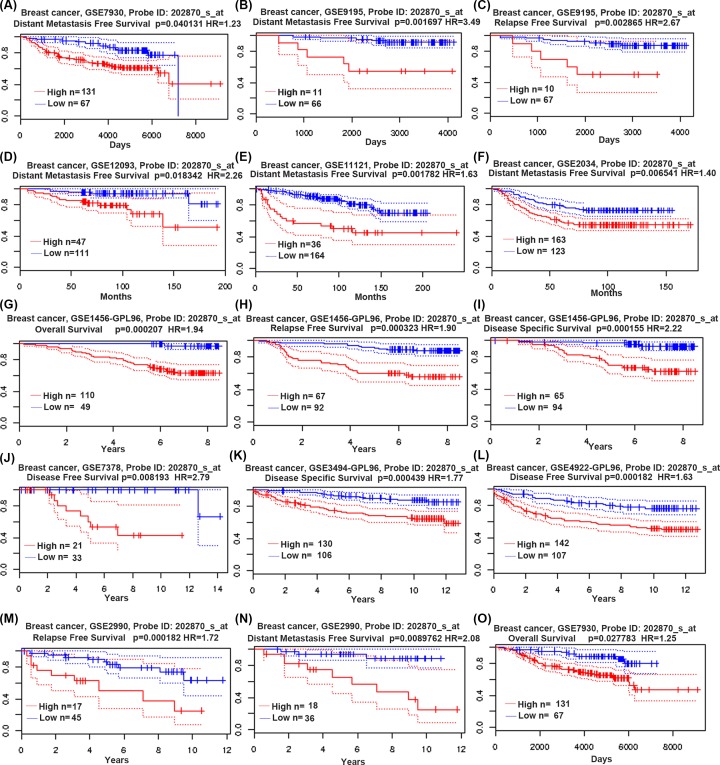
Survival curve evaluating the prognostic value of CDC20 Survival curve using the PrognoScan Analysis is shown for distant metastasis-free survival (**A,B,D**–**F,N**), RFS for different probes (**C,H,M**), disease-free survival (**J,L**) disease-specific survival (**I,K**) and OS (**G,O**). Red and blue stand for high and low expression of CDC20 gene, respectively.

Then, we investigated the prognostic value of CDC20 gene using the PrognoScan database. Breast cancer patients with lower expression of CDC20 (blue) significantly showed preferable distant metastasis-free survival (Figure 5A,B,D–F,N and Table 3). Reduced CDC20 level (blue) was related to better RFS (Figure 5C,H,M and Table 3) and the cases with increased CDC20 gene presented worse disease-free survival (Figure 5J,L and Table 3). Moreover, down-regulated CDC20 gene (blue) was strongly associated with better disease-specific survival (Figure 5I,K and Table 3) and up-regulated CDC20 gene (red) was related to worse OS (Figure 5G,O and Table 3).

**Table 3:** The association of CDC20 expression and the survival in breast cancer patients

Dataset	End point	Probe ID	Location	*n*	Cox *P*-value	HR (95% CI)
GSE7390	Distant metastasis-free survival	202870_s_at	Uppsala, Oxford	198	0.040131	1.23 (1.01–1.49)
GSE9195	Distant metastasis-free survival	202870_s_at	GUYT	77	0.001697	3.49 (1.60–7.60)
GSE9195	RFS	202870_s_at	GUYT	77	0.002865	2.67 (1.40–5.10)
GSE12093	Distant metastasis-free survival	202870_s_at	IO, NCI	136	0.018342	2.26 (1.15–4.43)
GSE11121	Distant metastasis-free survival	202870_s_at	Mainz	200	0.001782	1.63 (1.20–2.21)
GSE2034	Distant metastasis-free survival	202870_s_at	Rotterdam	286	0.006541	1.40 (1.10–1.79)
GSE1456-GPL96	OS	202870_s_at	Stockholm	159	0.000207	1.94 (1.37–2.74)
GSE1456-GPL96	RFS	202870_s_at	Stockholm	159	0.000323	1.90 (1.34–2.70)
GSE1456-GPL96	Disease-specific survival	202870_s_at	Stockholm	159	0.000155	2.22 (1.47–3.36)
GSE7378	Disease-free survival	202870_s_at	UCSF	54	0.008193	2.79 (1.30–5.98)
GSE3494-GPL96	Disease-specific survival	202870_s_at	Uppsala	236	0.000439	1.77 (1.29–2.44)
GSE4922-GPL96	Disease-free survival	202870_s_at	Uppsala	249	0.000182	1.63 (1.26–2.11)
GSE2990	RFS	202870_s_at	Uppsala, Oxford	62	0.014206	1.72 (1.11–2.65)
GSE2990	Distant metastasis-free survival	202870_s_at	Uppsala, Oxford	54	0.008976	2.08 (1.20–3.59)
GSE7390	OS	202870_s_at	Uppsala, Oxford	198	0.027783	1.25 (1.03–1.54)
GSE3143	OS	38414_at	Duke	158	0.010445	1.67 (1.12–2.41)
E-TABM-158	Disease-specific survival	202870_s_at	UCSF	117	0.038478	0.75 (0.57–0.98)

### Co-expression of CDC20 gene

**Figure 6:**
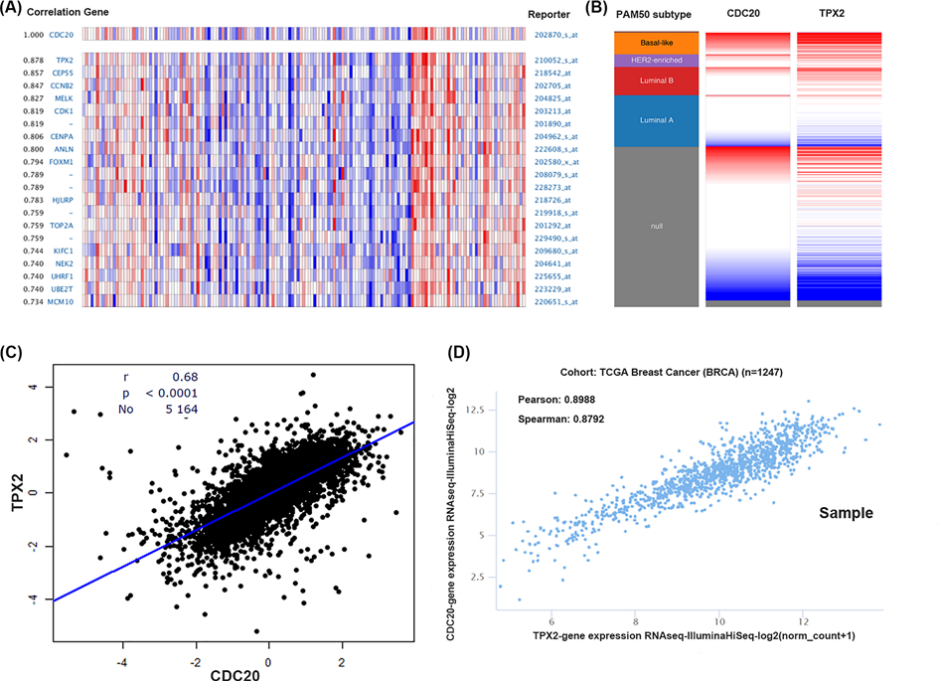
Co-expression of CDC20 (**A**) CDC20 co-expression of genes analyzed using Oncomine. (**B**) Relationship between CDC20 and TPX2 in breast cancer analyzed using bc-GenExMiner v4.0. (**C**) Heat map of CDC20 expression and TPX2 mRNA expression across PAM50 breast cancer subtypes in the TCGA database, determined using UCSC Xena; D: correlation between CDC20 and TPX2 mRNA expression in the TCGA database, determined using UCSC Xena.

Finally, we investigated the co-expression of CDC20 gene using the Oncomine database. The co-expression profile of CDC20 was identified with a large cluster of 17779 genes across 159 breast cancer samples (Figure 6A). Targeting protein for Xenopus kinesin-like protein 2 (TPX2) is a top correlated gene, which is a microtubule-associated protein and encoded by a gene located on human chromosome band 20q11.1 [22]. A positive correlation between CDC20 and TPX2 expression was revealed using bc-GenExMiner 4.2 (Figure 6C). Moreover, after analyzing breast cancer patient data in the TCGA database using the UCSC Xena web-based tool, again we confirmed a positive correlation between CDC20 and TPX2 expression, as shown in the heat map (Figure 6B,D). These results suggested that CDC20 might be closely related to the TPX2 signaling pathway in breast cancer.

## Discussion

Cell division cycle 20 (CDC20) is a vital molecule which acts as an important role in the cell cycle and an activator of the anaphase-promoting complex (APC/C) [23]. Higher expression of CDC20 has been observed in a variety of human cancers and is correlated with poor prognosis [7,24,25]. However, the significance of CDC20 expression in the development and prognosis of breast cancer remains largely unclear. To the best of our knowledge, this is one of the first study to identify CDC20 as a potential predictive biomarker for prognosis of breast cancer using comprehensive bioinformatics analysis.

In the present study, we performed a bioinformatics analysis of the clinical parameters and survival data related to CDC20 in breast cancer patients by pooling and analyzing several online tools. Comparing with normal tissues, Oncomine database revealed that CDC20 was expressed higher in different types of breast cancer including medullary breast carcinoma, invasive ductal breast carcinoma, invasive lobular breast carcinoma, and so on. CDC20 was also expressed higher in breast cancer tissues compared with adjacent normal tissues of patients in our hospital, confirming the results from databases online. Moreover, we also found patients with a more advanced stage of breast cancer tended to express higher levels CDC20. Consistently, Yuan et al. [26] reported that the mRNA and protein levels of CDC20 were significantly higher in breast cancer cells and high-grade primary breast cancer tissues.

For nodal status, there was no significant difference between positive and negative groups. ER and PR status were negatively correlated with CDC20 level. Conversely, SBR grade, NPI index, HER-2 status, basal-like status, and triple-negative status were positively related to CDC20 expression in breast cancer patients with respect to normal individuals. It is generally known that breast cancer patients with ER or PR positive, HER-2 negative, non-basal-like, or non-triple-negative status have a preferable outcome [27]. Therefore, these results indicated that lower expression of CDC20 may predict a better prognosis in breast cancer.

We further investigated the prognostic value of CDC20 in breast cancer using the PrognoScan database. These pooled results showed that higher CDC20 expression correlated with worse distant metastasis -ree survival, RFS, disease-free survival, disease-specific survival, and OS. These findings were in agreement with the notion of CDC20 as a tumor oncogene and a potential predictive biomarker for prognosis of breast cancer [13].

Finally, we checked the co-expression of CDC20 gene using the Oncomine, bc-GenExMiner, and UCSC Xena web-based tools and found that TPX2 was positively correlated with CDC20 expression. TPX2 is a microtubule-associated protein that is encoded by a gene located on human chromosome band 20q11.1 [22]. Overexpression of TPX2 has been observed in lung cancer, hepatic cancer, colon cancer [28–30]. Moreover, TPX2 is also a marker of poor tumor prognosis in several cancers [31]. Both CDC20 and TPX2 were related to the process of cell cycle [32]. Using bioinformatics analysis, Zhang et al. [33] found that elevated mRNA levels of CDC20 and TPX2 are associated with poor prognosis of lung adenocarcinoma. These observations, along with our findings of CDC20 in survival data, provided evidence that CDC20 gene might promote tumor progress associated with TPX2 expression.

In conclusion, the study was performed to comprehensively analyze the expression pattern, potential function, and distinct prognostic effect of CDC20 in breast cancer by pooling all currently available data online. CDC20 was highly expressed in different subtypes of breast cancer compared with normal tissues and was associated with several important clinical parameters. CDC20 could be considered as a potential predictive indicator for prognosis of breast cancer with co-expressed TPX2 gene. Over the past several decades, much research has focused on identifying new prognostic markers in order to make better clinical decisions and improve therapy and outcomes. More in-depth experiments are needed to validate the value of CDC20 for clinical decision-making in breast cancer.

## Abbreviations

bc-GenExMiner v4.2: Breast Cancer Gene-Expression Miner v4.2
CDC20: cell division cycle protein
ER: estrogen receptor
HER-2: epidermal growth factor receptor-2
NPI: Nottingham prognostic index
OS: overall survival
PR: progesterone receptor
RFS: relapse-free survival
RT-PCR: quantitative real-time PCR
SBR: Scarff–Bloom–Richardson
TCGA: The Cancer Genome Atlas
TPX2: Targeting protein for Xenopus kinesin-like protein 2
